# A Manual of Surgical Treatment

**Published:** 1902-03

**Authors:** 


**A Manual of Surgical Treatment.** By W. WATSON CHEYNE and F. F. BURGHARD. Part V. Pp. xxii., 470. London: Longmans, Green and Co. 1901.

As we have already reviewed four of these volumes, we need say nothing further as to the scope and merits of the work. Only the first half of this volume is by Mr. Watson Cheyne and Mr. Burghard; the second half, which deals with diseases of the nose, throat, and larynx, is written by Dr. Lambert Lack. The chapter on the operative treatment of cancer of the larynx is, however, written by the original authors. The first half of the volume is occupied by affections of the head, face, jaw, and those of the larynx and trachea, which come under the care of the general surgeon. The treatment of some diseases about which we find very little information in the ordinary text-book—such as tuberculous disease of the skull—is fully described, but the treatment of some other diseases seems hardly to have received sufficient attention. No doubt the ground covered is a very large one, and even in six volumes it is very difficult to go very fully into the treatment of all the important diseases and injuries.

The complete operation for chronic middle-ear suppuration is clearly described by Dr. Lack. It consists in a free opening into the antrum, and from the antrum into the auditory meatus, together with the removal of the ossicles, and curetting the tympanum.

We regret that the only method of draining the distended ventricles and subarachnoid space described, is the one in which the posterior fossa is trephined, and the space above the fourth ventricle opened. The authors write of it as the best method for drainage of the posterior part of the cranial cavity; but why should they describe only a method for draining this part? They write that the great difficulty is to maintain the drainage, "as a tube of medium size gets blocked, and if large allows the semi-fluid cerebellum to escape." This difficulty is not present if the lateral ventricle is drained through the cerebral cortex, and the danger of injury to the fourth ventricle is avoided.

It is very interesting to read Mr. Watson Cheyne's latest expression of opinion with regard to the results of the operation which he has introduced of internal drainage of the ventricle in hydrocephalus. It is not encouraging. He finds in congenital cases, although the distention of the head disappears, the children mostly die in a few months, and in the acquired cases the operation has not been successful in establishing a communication between the ventricle and the subdural space. He proposes to enclose the strands of catgut in a decalcified bone drainage-tube, in these cases.

